# Following up patients with depression after hospital discharge: a mixed methods approach

**DOI:** 10.1186/1752-4458-5-28

**Published:** 2011-11-10

**Authors:** Franciska A Desplenter, Gert J Laekeman, Steven R Simoens

**Affiliations:** 1 Research Centre for Pharmaceutical Care and Pharmaco-economics, Faculty of Pharmaceutical Sciences, Katholieke Universiteit Leuven, ON2 Herestraat 49 P.O.Box 521, 3000 Leuven, Belgium; 2 Psychiatric Hospital Sint Jan, Eeklo, Belgium

## Abstract

**Background:**

A medication information intervention was delivered to patients with a major depressive episode prior to psychiatric hospital discharge.

**Methods:**

The objective of this study was to explore how patients evolved after hospital discharge and to identify factors influencing this evolution. Using a quasi-experimental longitudinal design, the quantitative analysis measured clinical (using the Hospital Anxiety and Depression Scale, the somatic dimension of the Symptom Checklist 90 and recording the number of readmissions) and humanistic (using the Quality of Life Enjoyment and Satisfaction Questionnaire) outcomes of patients via telephone contacts up to one year following discharge. The qualitative analysis was based on the researcher diary, consisting of reports on the telephone outcome assessment of patients with major depression (n = 99). All reports were analyzed using the thematic framework approach.

**Results:**

The change in the participants' health status was as diverse as it was at hospital discharge. Participants reported on remissions; changes in mood; relapses; and re-admissions (one third of patients). Quantitative data on group level showed low anxiety, depression and somatic scores over time. Three groups of contributing factors were identified: process, individual and environmental factors. Process factors included self caring process, medical care after discharge, resumption of work and managing daily life. Individual factors were symptom control, medication and personality. Environmental factors were material and social environment. Each of them could ameliorate, deteriorate or be neutral to the patient's health state. A mix of factors was observed in individual patients.

**Conclusions:**

After hospital discharge, participants with a major depressive episode evolved in many different ways. Process, individual and environmental factors may influence the participant's health status following hospital discharge. Each of the factors could be positive, neutral or negative for the patient.

## Background

"Seamless care" is the continuity of care delivered to a patient in the health care system across the spectrum of caregivers and health care settings [1]. This delivery of care should be consistent with the ongoing needs of the individual patient. In practice, several breaks or practice gaps can occur in the continuity of patient care. The Continuity of Care Task Force of the American Society of Health-System Pharmacists identified eight categories of such practice gaps: clinical gaps, patient gaps, communication gaps, organizational gaps, coordination gaps, professional gaps, policy gaps and technology gaps [1]. It is a challenge to set up programs and initiatives to overcome these gaps in order to obtain continuity of care for every patient [2-5].

The provision of seamless care is especially relevant in the psychiatric setting. Discharge of patients from a psychiatric hospital is a critical point as this may be a risk for discontinuity of care. Psychiatric patients frequently experience serious symptoms and demonstrate disturbed behaviours (e.g. regressive functioning) in the very early post-discharge period. The very early post-discharge period was defined as the very first days after discharge. Wells explained that symptom exacerbations immediately after discharge are likely to be related to multiple intrapsychic and environmental determinants that make patients vulnerable to being temporarily overwhelmed by the challenges of adjusting to separation from a supportive hospital and reunion with a potentially less hospitable world [6]. Wells focused only at the early post-discharge period and not on the longer post-discharge period. Little literature is available on long term data after hospital discharge for patients with major depression [7], except from some studies on suicidal ideation [8,9].

A review on educational medication interventions in a psychiatric population showed that studies with heterogeneity in settings, interventions and target population has been performed. The main outcomes were compliance and knowledge for which both improvements after the educational medication intervention were seen [10]. The GIPPOZ-study [11,12] was set up to explore specifically the impact of a medication information intervention on antidepressants prior to discharge from a psychiatric hospital. GIPPOZ is a Dutch acronym for 'Differentiated Information for Psychiatric Patients at Hospital Discharge'. This was a clinical pharmacy intervention aiming at seamless or continuity of care by focusing on the patients' pharmacotherapy. In addition to examining the impact of the medication information intervention, a telephone diary was completed by the main researcher to explore patients' status after discharge by giving answers to the questions: how did patients proceed after discharge? Which factors contributed to patients' status?

The aim of the study was to answer the following research questions. (1) How do quantitative indicators for clinical and humanistic outcomes change up to three months after hospital discharge? (2) What does the telephone diary tell about the qualitative evolution of the patients' (mental) health status? (3) Which factors are contributing to this evolution? (4) To what extent new research questions can be formulated based upon the needs expressed by the patients during the telephone contacts?

## Methods

### Data sources

Patients were eligible for inclusion in the GIPPOZ-study [11,12] if they were at least 18 years old, if they had a major depressive episode as primary diagnosis according to the DSM-IV-TR criteria, it they took at least one antidepressant, if they were Dutch-speaking, if they could be reached by telephone for follow-up and if the treating psychiatrist confirmed these inclusion criteria. The GIPPOZ-study is a longitudinal study in 11 Flemish psychiatric hospitals. A quasi-experimental design was applied. It comprised three study groups: one control group (usual care) and two experimental groups (undifferentiated and differentiated information intervention depending on patients' information desire). Each hospital was assigned to one of the three study groups. Outcomes assessed in the GIPPOZ-study were compliance, depressive symptoms, somatic symptoms, side effects, costs of medicines and health care professionals, number of work days lost, quality of life and satisfaction.

Ninety-nine patients with a primary diagnosis of major depression according to the DSM-IV-TR criteria [13] were included in the GIPPOZ-study. Outcomes of the GIPPOZ-study were assessed via telephone contacts at four different points in time: shortly after the intervention (within one week after the intervention) (n = 96), one month after discharge (n = 89), three months after discharge (n = 80) and one year after discharge (n = 78). In total, 21 patients dropped out due to lost to follow-up and due to the fact that some patients did not longer want to participate. At inclusion, patients received a booklet containing all questionnaires for the four contact times to facilitate the outcome measurements.

Quantitative data on outcome measures were collected by means of questionnaires as reported by patients during telephone follow-up contacts by the main researcher. Data related to anxiety and depressive symptoms (Hospital Anxiety and Depression Scale (HADS))[14], somatic symptoms (somatic subscale of the Symptom Checklist 90 (SCL))[15], quality of life (Quality of Life Enjoyment and Satisfaction Questionnaire (Q-LES-Q))[16] on the first three follow-up contacts. One year after discharge, readmission and use of antidepressant(s) were self-reported.

Notes were made during the telephone contacts of the patient's comments, questions and spontaneous stories. A qualitative report was written on each telephone contact in the GIPPOZ researcher diary. This researcher diary was a supportive instrument for future telephone contacts as the researcher used this diary to keep track of the issues raised in each contact with each patient. All 343 reports were included in the analysis [17]. The reports are primary source documents with a private, study status. All data were anonymised. Ethical approval was obtained for the GIPPOZ-study and participants provided written consent at the start of the study.

To address the aims of the current study, a mixed methods analysis was conducted.

### Quantitative data analysis

Descriptive statistical analyses were performed to describe patient sample (mean and standard deviation for normally distributed variables, median and percentiles for non-normally distributed data, and frequencies) and measurement scores (mean and standard deviations). As the aim of the current study is to assess how patient evolve after discharge, data of the GIPPOZ-study was pooled in one group. To assess differences in measurement scores (HADS, SCL and Q-LES-Q) over time, a linear model for repeated measures using an unstructured covariance matrix was applied. Analyses were performed in SPSS 16.00 for Windows (SPSS Inc., Chicago, IL).

### Qualitative data analysis

The researcher diary was content analyzed using a qualitative approach. This qualitative analysis was performed inductively. The reports in the telephone diary were examined to identify the content of what patients told, using thematic framework analysis [18]. Framework analysis was applied because it provides structure and coherence to the large amount of data as the GIPPOZ researcher diary counts more than 200 pages. Second, it facilitates systematic analysis. And finally, it relies on the creative and conceptual ability of the analyst to make sense of the content [19]. Thematic framework analysis consists of five stages as was described by Pope and Mays: (I) familiarization (reading of the reports), (II) identifying a framework, (III) indexing, (IV) charting and (V) mapping and interpreting [20]. The software QSR NVivo 7 (QSR International Pty Ltd., 2006) was used to facilitate data management.

A thematic framework was built on consensus between the main researcher and the supervisors of this study and was based on issues emerging from the data. The researcher diary was indexed and analyzed by the main researcher. When citing quotes of patients, the patients are referenced by including the unique number of the patient in the GIPPOZ-study. *Verbatim *citations of patients are formatted in italic, while notes of the diary are formatted regular.

## Results

### Sample characteristics

A summary of demographic characteristics of all included participants in the GIPPOZ-study (n = 99) is given in Table 1. The majority of the participants were female (63%) with a mean age of 46 years. The median length of stay at the psychiatric hospital was 60 days. Half of the participants had a severe without psychotic symptoms depression at hospital admission. Two thirds of the participants had a psychiatric co-morbidity on top of the main diagnosis of major depression.

**Table 1:** Demographic characteristics of the GIPPOZ study sample (n = 99)

Demographic characteristic	N (%)
Female gender	62 (62.6%)

Age *(mean ± SD) (in years)*	46.1 ± 11.1

Education	
*Low education (till secondary school)*	68 (68.7%)
*Higher education*	31 (31.1%)

Occupation	
*Blue collar worker*	38 (38.4%)
*White collar worker*	45 (45.5%)
*Other*	16 (16.2%)

Maritial status	
*Single (single, divorced or widow(er) without partner)*	43 (43.4%)
*Living together (married or living together)*	56 (56.6%)

Severity of major depression at admission	
*Mild*	11 (11.1%)
*Moderate*	31 (31.3%)
*Severe without psychotic characteristics*	48 (48.5%)
*Severe with psychotic characteristics*	9 (9.1%)

Psychiatric comorbidity	
*Anxiety*	23 (23.2%)
*Substance related disorder*	19 (19.2%)
*Other*	25 (25.3%)
*None*	32 (32.3%)

Presence of a somatic comorbidity	65 (65,7%)

Age at first onset *(mean ± SD) (in years)*	36.2 ± 12.6

Number of previous admissions for depression in lifetime (*Median [25;75 percentile])*	2.0 [1.0;3.0]

Length of hospital stay (*Median [25;75 percentile])*	60.0 [31.0;98.0]

Number of medicines related to Central Nervous System at hospital discharge (*Median [25;75 percentile])*	3.0 [2.0;4.0]

### Quantitative data

Follow-up measurement scores of anxiety, depressive (HADS) and somatic (SCL) symptoms and quality of life (Q-LES-Q) are provided in Table 2. Presence and severity of symptoms was low. Quality of life was scored as 62-65% of maximum possible. Satisfaction with medicines was higher (76-78%).

**Table 2:** Anxiety, depressive and somatic symptoms and quality of life measurements during GIPPOZ follow-up (at discharge, one month and three months after discharge)

Measurement	At discharge	One month after discharge	Three months after discharge	P-value
HADS anxiety ^a^	8.6 ± 4.3 (n = 96)	9.3 ± 4.5 (n = 89)	8.9 ± 4.8 (n = 80)	NS

HADS depression ^a^	7.1 ± 4.6 (n = 96)	8.3 ± 5.1 (n = 89)	7.8 ± 4.9 (n = 80)	P = 0.033

SCL somatic dimension ^b^	22.9 ± 9.1 (n = 96)	24.3 ± 8.9 (n = 89)	23.6 ± 9.3 (n = 80)	NS

Q-LES-Q General activities ^c^	68.7 ± 41.4 (n = 96)	65.8 ± 14.3 (n = 89)	65.9 ± 15.0 (n = 80)	P = 0.049

Q-LES-Q Satisfaction with medicines ^d^	78.3 ± 15.0 (n = 96)	76.1 ± 14.3 (n = 87)	75.9 ± 12.6 (n = 79)	NS

Q-LES-Q Overall quality of life ^d^	68.5 ± 20.5 (n = 96)	62.7 ± 20.9 (n = 89)	65.0 ± 22.4 (n = 80)	P = 0.034

HADS = Hospital Anxiety and Depression Scale; SCL = Symptom Checklist; NS = not significant; Q-LES-Q = Quality of Life Enjoyment and Satisfaction Questionnaire

^a ^7 items, 4 point scale from 0 to 3 for each item, total score is sum of the scores on each of the 7 items, range of total score is 0-21, score interpretation: non-cases for anxiety/depression (≤ 7), doubtful cases for anxiety/depression (8-10) and definite cases for anxiety/depression (≥11)

^b ^12 items, 5-point Likert scale (score from 1 to 5), total scores is sum of the scores on each of the 12 items, range of total score is 12-60, the higher the score the more the patient was hindered by somatic symptoms

^c ^14 items, 5-point Likert scale (score from 1 to 5), score is expressed as the percentage of the maximum score, the higher the score the more satisfied the patient was

^d ^1 item, 5-point Likert scale (score from 1 to 5), score is expressed as the percentage of the maximum score, the higher the score the more satisfied the patient was

One year after discharge, 34 (43.6%) out of 78 participants had no changes in their antidepressant pharmacotherapy. Eight participants (10.3%) stopped all their antidepressants due to intentional non-compliance and due to instruction by the prescribing physician. Changes in the antidepressant pharmacotherapy were made for 38 patients (56.4%): dose change (n = 8), switch of antidepressant (n = 8), additional antidepressant (n = 8) or fewer antidepressants (n = 12).

**Figure 1:**
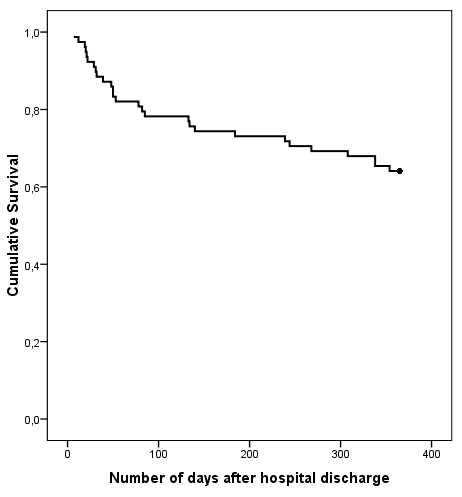
**A Kaplan Meier graph representing the proportion of patients readmitted and the time interval (expressed in days) between hospital discharge (day 0) and first readmission**.

At one year after discharge 28 out of 78 participants (36%) were readmitted to a psychiatric hospital. In total, 49 readmissions occurred. 35 out of the 49 readmissions were reported to be related to the depression pathology. A Kaplan Meier graph (Figure 1) was plotted to illustrate the proportion of participants readmitted and the time interval (expressed in days) between hospital discharge and first readmission: the majority of the participants were readmitted within 100 days after discharge.

### Qualitative data

The change in the participants' health status was as diverse as it was at the start of the GIPPOZ-study. Some participants continued to improve without having symptoms of low mood. Other participants reported on having ups and downs in several degrees of severity. Another group of participants had again more and more depressive symptoms which, in some cases, led to a relapse and even to a readmission to a psychiatric hospital because of e.g. a crisis situation, suicide attempt, fear of escaping into alcohol. A whole spectrum of evolutions in participants' health status has been observed. The diversity present at the start of the GIPPOZ-study was also seen at the end of the GIPPOZ-study (one year after their discharge from hospital). *Quotes are provided in *Table 3.

**Table 3:** Illustrative quotes for each section of the results

Section results	Illustrative quotes
Diversity in health status	'In February 2008, the patient was readmitted because she was not able to see her way out. She had enough of it. She was readmitted on a full time basis for a period of 5 months. Afterwards, she went to day care therapy. After two weeks of day care, she made a suicide attempt. This resulted immediately to a new full time readmission. During that readmission, she kept low profile because she planned to have a holiday. She would love to leave for her holiday.' *(GIPPOZ 36)* *''I feel well but I have to fight against it every day. It is a daily struggle not to give in to depression.' *As far as the rest is concerned, the patient manages herself.' *(GIPPOZ 67)*

Change over time	*''Actually, it goes quite well. One day goes better than the other day. Today for example, if you have a look outside the window ... it has been raining all day long.' *The patient asks me what the weather is like in Leuven.' *(GIPPOZ 17)* 'The patient adds that these changes are present within the same day rather than being different between days.' *(GIPPOZ 81)*

Self-caring process	*''At home, the expectations are high ... I manage but I sleep a lot. It has to go quickly once you are home again. Some things seem simple for my surroundings but for me it is quite a task.'' (GIPPOZ 61) (globally neutral appreciation)* 'The transition from being admitted to home was experienced as very difficult by the patient. That should not be underestimated. She was also very emotional. Taking care of her (shopping, cooking, etc.) was all right. The most difficult thing was to fill in her time. She had too much time ... and started to worry. She needed to structure her life again. On days of day care, it was all right. The other days were more difficult for the patient.' *(GIPPOZ 87) (globally neutral appreciation)*

Medical care	'Day care was organized for this patient. She never went because the patient needed to pay € 100 every month for this service.' *(GIPPOZ 2) (negative appreciation)* 'She visits her psychiatrist every six weeks. If needed, she can visit the psychologist in hospital. She is followed up well. The patient indicates that this follow-up is a positive thing. In the past, this was not the case and she did not like that.' *(GIPPOZ 87) (positive appreciation)*

Return to work	'After hospital discharge, the patient followed 14 days of day care. He liked this very much as steppingstone. Because at discharge, everything is coming up to you and you wonder if you will manage. Day care took this aspect very well into account. The hospital discouraged the patient to go back to work immediately because the transition would be too big. After 14 days of day care, the patient went back to work. This transition passed smoothly.' *(GIPPOZ 6) (positive appreciation)* 'The pressure was too high after discharge. He had to work again full time and it had to go too fast. If he would had get more time to build up quietly, he thinks he would have managed.' *(GIPPOZ 92) (negative appreciation)*

Daily living	*''Life is simple and everything is well structured at the hospital ward. When I was on day care, it could not cope ... I even didn't eat any more. It was no go.'' (GIPPOZ 82) (negative appreciation)* 'The structure is not yet what it should be. Therefore, since two or three months, the psychiatric homecare passes by every two weeks for an hour. They try to evaluate how the patient is managing and try to bring some structure in her daily life. They ask if she is taking her medicines, if she takes care of herself ...' *(GIPPOZ 89) (positive appreciation)*

Symptom control	'When the patient feels bad, she calls 106 (= suicide prevention and support telephone helpline). Feeling bad means to her that she has dark and suicidal thoughts. The patient has still such ideas. In hospital, they told her that she will need to cope with those ideas. The patient hopes that one day it will be better or that she won't have those ideas any more. That way, her life would be better and more meaningful.' *(GIPPOZ 44) (negative appreciation)* 'It goes quite well. She tells that she is suffering quite a lot of her back for the moment (bilateral facet problem; discus problem and calf back). She visits a physiotherapist twice a week. According to the patient, the terrible back ache is associated with the stress of the past months.' *(GIPPOZ 47) (positive appreciation)*

Medication	'The patient has his own thoughts about the use of antidepressants. He compares them with pain killers for toothache. *'The pain killers won't make the cavity in your tooth go away. They do not take away the cause.'' (GIPPOZ 74) (neutral, appreciation)* *''I don't take my antidepressants any more ... they can't get in any more. I took them several years. I become crazy of it.' *He manages fairly to take his cardiovascular medicines.' *(GIPPOZ 69) (negative appreciation)* 'This summer, she stopped taking her medication because she felt better. Later, she relapsed. She forgot a consultation with her psychiatrist during holiday. She won't do that anymore in the future.' *(GIPPOZ 33) (positive appreciation)*

Personality	*''Looking forward, not backward.' *The patient learned to life according to what his body is saying.' *(GIPPOZ 26) (positive appreciation)* *''I'm feeling good. I'm a bit perfectionist. My wife warns me not to overdo myself.'' (GIPPOZ 51) (positive appreciation)*

Finances	'The patient tells she cannot take care of herself. She spends too much money through which she gets in trouble all over again.' *(GIPPOZ 63) (negative appreciation)* *''Actually, I should visit the cardiologist and the dentist ... but yeah, that's all very expensive ...' *Necessary care is postponed due to financial constraints.' *(GIPPOZ 88) (negative appreciation)*

Unable to work/no job	'Because of her back surgery and because she could not resume work after a certain period, the patient was sacked. The patient does not have any diplomas. She cannot do heavy work anymore. The patient is in a very difficult situation.' *(GIPPOZ 59) (negative appreciation)* 'Things go well at work. The patient is very happy about that. She considers her work in another way: she is much more open. She liked her job very much before but now, she likes it even more and now she knows this is really what she wants. It gives her energy.' *(GIPPOZ 91) (positive appreciation)*

Social relations	*''Social relations are one of my difficulties'*, the patient tells. She says she is closing herself for everyone. This is definitely a task for her.' *(GIPPOZ 44) (negative appreciation)* 'The patient feels not understood by his family, even by his daughter. He cannot count on their understanding because they do not know what it means to suffer from depression. The patient admits he also could not get it right in the past. The patient tries to explain but that does not always work out. The family cannot imagine their selves well ... ' *(GIPPOZ 26) (negative appreciation)* *''The leisure activities are good thanks to a friend of mine who drags me everywhere.'' (GIPPOZ 89) (positive appreciation)*

Leisure activities	'The patient likes to draw, to paint and to read. But when she wants to do these things, she looks around and she sees the household work. Then she has a feeling of guilt. She does not succeed in relaxing. She gives priority to the chores.' *(GIPPOZ 19) (negative appreciation)* 'The patient had the prospect of doing walks with young children. These walks were fantastic. He really enjoyed these walks.' *(GIPPOZ 81) (positive appreciation)*

Home	'After the last admission, she did not return to home. In hospital, she arranged a home in Ostend where she went straight away after her discharge. She took a new start over there. She had enough of it and did not like to return to her husband. In the mean time, they divorced. She feels much more peaceful now.' *(GIPPOZ 88) (positive appreciation)* 'The patient lived together with his son in a flat. Now, his son is going to life together with his girlfriend. So, he has to move now because he cannot afford this flat anymore.' *(GIPPOZ 29) (negative appreciation)*

About one third of the participants reported that their mood changed from day to day. Changes in how they felt were also reported within the same day. *Quotes are provided in *Table 3.

In the next paragraphs, the themes that emerged from the data are presented. These themes consist of factors reported during the telephone contacts. The identified factors contributed to the participants' current status of health and well-being. These factors can be divided in three groups: process factors, individual factors and environmental factors. A factor can ameliorate or deteriorate or just be neutral for the status of a participant. As many factors contribute to the overall participants' status, change in one factor might not be directly linked to a change in the overall participants' status. In most participants, a mix of positive, negative and neutral factors was observed. An appreciation is included after each citation.

### Process factors

#### Self caring process

Many participants told to be happy to be able to go back home. This was observed in participants with either a short or a long hospital stay. Some of them wondered how this would proceed and were curious how they would manage. Other participants did not have worries about the transition between hospital and home. The transition itself was experienced as difficult in some cases as participants needed to re-adapt to the home environment after being living in the structured environment of a hospital. In other cases this transition proceeded smoothly. This was often mentioned by participants for whom this transition was not abrupt but rather step-down e.g. by following day care after discharge. *Quotes are provided in *Table 3.

#### The patient and the caring system

Medical care after discharge was available for the majority of the participants. This was organized via day care, ambulatory consultations with the psychiatrists, psychologist consultations, general practitioner consultations or via an outpatient mental health centre. For some participants, medical follow-up was problematic due to several reasons: in search for another health care professional, participant had no time for the consultation, no money to pay the consultation, hospital/health care professional fully booked or participants did not wish to have medical care anymore. *Quotes are provided in *Table 3.

Getting back to work was another challenging process for participants. Timing for this process was very variable among participants. Some of them started to work again soon after discharge, whilst others returned to work after several months. Some of them started on a full time basis, whilst others used a step-up approach by starting on a part time basis. For some of them working was just as it was before or even better and for others it demanded a great adaptation and sometimes it was still too early to get back to work and needed an extension of their sick leave. Other participants did not manage to return to work within one year after discharge. *Quotes are provided in *Table 3.

Managing their own life and running their housekeeping was as challenging as getting back to work. Participants reported they had to adapt themselves again after this period of hospital stay. Sometimes it went smoothly and at other times it was a struggle to bring in some structure. *Quotes are provided in *Table 3.

### Individual factors

#### Symptom control

Except for a minority of the participants, presence of somatic symptoms was reported. These were gastro-intestinal problems, back problems, a recent surgery, headache and vague symptoms (trembling, tingling, equilibrium disturbances, dizziness and feeling hot or cold). Also psychiatric symptoms were reported: depressive symptoms including suicidal ideas, anxiety and substance related problems (alcohol, medicines or drugs). Some participants could dissociate somatic health from their mental health. *Quotes are provided in *Table 3.

#### Medication

Pharmacotherapy with antidepressants was present for all participants at hospital discharge. Some participants reported on a having a long search to find the antidepressant that works for them. Antidepressants were stopped, were changed and were started again. Several motivations were provided: antidepressant was no longer needed, did not work or caused too much side effects. Compliance was another issue for pharmacotherapy: having a relapse due to an early stop of the antidepressant was a strong motivator to be compliant to the antidepressant pharmacotherapy this time. Several participants reported that although the antidepressant is supportive, it is not a solution for the underlying problem(s) or for the cause of the depression. *Quotes are provided in *Table 3.

#### Personality

Participants had their own personality, way of living and coping styles. This can facilitate or complicate certain situations e.g. going through a difficult time. *Quotes are provided in *Table 3.

### Environmental factors

#### Material environment

Financial difficulties have been reported. Debt mediation was often used as a resource to get out of debt. This was seen as a support for the participants because they did not manage on their own. Because of these financial constraints, medical care was sometimes limited to essential care only and it also complicated daily life. *Quotes are provided in *Table 3.

Being unemployed, being disabled or not yet able to return to work were experienced as disappointments. This situation was often accompanied with a lower financial income. Dissatisfaction was sometimes present. For other participants, getting back to work turned out better than was expected. *Quotes are provided in *Table 3.

#### Social environment

Social relations with family and friends were sometimes reported to be difficult or complex: divorce, decease, illness, feeling lonely, lack of understanding on depression and objections towards antidepressant pharmacotherapy. Sometimes, the participant was not ready yet to take up again social life. Other participants were well supported by their environment. *Quotes are provided in *Table 3.

Leisure activities could be relaxing. Other participants did not have or did not desire leisure activities. Finding relaxation was regularly found to be difficult. *Quotes are provided in *Table 3.

Some participants reported to have difficulties in finding an appropriate home or not feeling well in their current home. Several participants moved. Some of them started all over again. *Quotes are provided in *Table 3.

## Discussion

This study has followed patients with a major depressive episode up to one year following discharge from a psychiatric hospital. The quantitative analysis indicated that some participants continued to experience depressive and somatic symptoms, had an impaired quality of life following hospital discharge, or were re-admitted to hospital. The literature indicates that clinical and psychosocial factors are predictors of outcomes or relapse of major depressive episodes. Clinical predictors included baseline depression severity, number and length of previous depressive episodes, age at first onset, previous ineffective treatment, absence of early response and increased number of co-morbidities [21-23]. Psychosocial predictors were lack of employment, low socio-economic status, marital status, poor functional status, expressed emotion, stress, disability and absence of social support [21,24,25]. A number of these factors were also identified in our qualitative research in the groups 'individual factors' and 'environmental factors'. Future research will need to clarify if there is a link between all of the identified factors and participants' status or outcomes. Further research is needed to study the identified factors more in depth and how they relate to each other in a qualitative and quantitative approach.

The current study did not report on the specific numbers that each factor was mentioned in the qualitative part. This has been controversial and is still a topic of debate. Maxwell listed the advantages and problems in using numbers in qualitative research [26]. The main reason why no numbers were reported is that numbers can lead to the inference of greater generality for the conclusions than is justified. As this study reports on spontaneous reports rather than on data collected in systematic interviews on all factors described in the results, such inferences would not be appropriate.

One third of the patients had a readmission of which the majority occurred in the first 100 days after hospital discharge. Direct comparisons with other countries are difficult due to important differences in the structure and organisation of the health care systems between countries. A Canadian study showed that 19% of the patients with depression were readmitted within one year [27]. The study showed also that readmission within 30 days were more likely among unemployed persons and were showing more depressive symptoms.

Risk of relapse has been associated with the presence of residual symptoms [28]. Both the quantitative and qualitative analyses showed that several participants experienced such residual symptoms. Keller suggested several approaches to increase the likelihood of remission. These were performing outcome measurement, optimization of dose, selection of antidepressant and pharmacologic adjuncts, optimization of the acute phase and attention to the three phases of treatment (acute, continuation and maintenance) [29]. The approaches listed by Keller are mainly related to pharmacotherapy. In clinical practice and especially in a hospital environment, the focus needs to be broader in order to improve patient care and outcomes. Attention for the clinical factors is as important as the psychosocial and process-related factors.

A whole spectrum of evolutions in participants' health status and well-being has been observed in the quantitative and qualitative analyses. Although process factors, individual factors and environmental factors influenced participant status following hospital discharge, we wish to emphasize that each individual participant had its own specific situation. Some of these factors were encountered as difficulties, others as neutral items and some as stimulators. This appreciation of factors fits with the transactional functional communication model of Barnlund. In this model, the patient as communicator has a central role. The patient assigns a certain meaning to cues and (s)he encodes perceptions and feelings into cues. These cues can be public (observable for everyone; e.g. participant has a plaster for his/her broken arm), private (not observable for everyone; e.g. financial problems) and behavioural non-verbal ones (e.g. closing eyes during conversation). Persons formulate an appreciation of each of these cues: neutral (will be filtered away) or negative or positive (both will strengthen the emotions) [30,31]. In the presentation of the results, an appreciation of the cue/factor was added to each quote. Future research could study if there are any patterns present between the type of cue/factor and the appreciation given to it.

In this study, a combination of quantitative and qualitative methods was applied because they are complementary to each other. Whereas the quantitative analysis was concerned with questions about the frequency of outcomes following hospital discharge, the qualitative analysis allowed us to gain in-depth knowledge on the experiences of participants. However, the analyses suffered from a number of limitations. First, not all participants completed the GIPPOZ-study. However, only one-fifth of participants dropped out of the study over a period of one year. This may be due to the specific method of following up performance, i.e. via telephone contact. Second, as the telephone follow-up conversations were not tape recorded, citations of participants included in the report were not *verbatim *but were based on the notes of the researcher. Third, not all participants were talkative. Some of them were more closed. Consequently, the reports of such participants were shorter and contained less detail on their situation. This is due to the standard procedure followed: the researcher was just asking the questions from the questionnaires. Participants were free to add any comments but were not asked to do so. If this would have been performed in a structured way for every participant, it would have been artificial and maybe some themes would not be identified. Fourth, no fixed structure was used for writing the reports. The only concern was to have a factual report of the conversation. Fifth, the results need to be seen in the context of post-discharge of patients with major depression. Sixth, no inference can be made on the possible influence of comorbidities on the outcomes of the GIPPOZ-study.

## Conclusions

This quantitative and qualitative analysis of patients with a major depressive episode following hospital discharge highlighted the continued presence of depressive and somatic symptoms, impaired quality of life and the need for re-admission in some participants. Although process factors, individual factors and environmental factors may influence participant status following hospital discharge, participants differed in how they appreciated these factors as difficulties, neutral items or stimulators. It needs to be emphasized that each individual participant had its own specific situation.

## Competing interests

The authors declare that they have no competing interests.

## Authors' contributions

FAD designed the study and wrote the protocol under supervision of GJL and SRS. GRG was consulted for the design of the study and was responsible for patient recruitment; data collection during intervention and data collection on patients' readmissions. FAD undertook the quantitative and qualitative analysis. FAD, GJL and SRS built the thematic framework used in the qualitative analysis and discussed the analyses. All authors contributed to and have approved the final manuscript.
